# Analysis of procalcitonin and CRP concentrations in serum of patients with chronic spontaneous urticaria

**DOI:** 10.1007/s00011-012-0580-1

**Published:** 2012-12-04

**Authors:** A. Kasperska-Zajac, A. Grzanka, E. Machura, B. Mazur, M. Misiolek, E. Czecior, J. Kasperski, J. Jochem

**Affiliations:** 1 Chair and Clinical Department of Internal Diseases, Allergology and Clinical Immunology, Medical University of Silesia, ul. Ceglana 35, 40-952 Katowice, Poland; 2 Department of Internal Diseases, Dermatology and Allergology, Medical University of Silesia in Katowice, Katowice, Poland; 3 Chair and Department of Pediatric in Zabrze, Medical University of Silesia, Zabrze, Poland; 4 Department of Microbiology and Immunology, Medical University of Silesia, ul. Jordana 19, 41-808 Zabrze, Poland; 5 Chair and Clinical Department of Otolaryngology in Zabrze, Medical University of Silesia, Katowice, Poland; 6 Department of Prosthetic Dentistry in Bytom, Medical University of Silesia, Katowice, Poland; 7 Department of Basic Medical Sciences, Medical University of Silesia, ul. Piekarska 18, 41-902 Bytom, Poland

**Keywords:** Procalcitonin, Chronic urticaria, Acute phase response, C-reactive protein

## Abstract

**Background:**

Our previous findings showed the importance of analysing the peripheral markers of acute phase response (APR) activation, C-reactive protein (CRP) and IL-6 in the context of urticaria activity and severity. However, these biomarkers do not reliably differentiate between APR to infectious and the disease severity.

**Aim:**

In order to investigate a possible association between the immune-inflammatory activation markers CRP and procalcitonin (PCT).

**Methods:**

Serum PCT and CRP concentrations were measured in patients with CU of varying severity as well as in healthy subjects.

**Results:**

Serum PCT and CRP concentrations were significantly increased in more severe CU patients when compared to healthy controls and mild CU, and within the CU population there was a significant correlation between concentrations of PCT and CRP. Serum PCT concentrations remained within normal ranges in most CU patients and were only slightly elevated in some severe CU cases.

**Conclusions:**

PCT serum concentration may be only slightly elevated in some cases of severe CU. Upregulation of PCT synthesis accompanied by parallel changes in CRP concentration reflects a low-grade systemic inflammatory response in CU. PCT should be considered as a better marker than CRP to distinguish between APR to infection and an active non-specific urticarial inflammation.

## Introduction

Spontaneous chronic urticaria (CU) can be identified as a mast cell- and basophil-dependent inflammatory disorder of the skin accompanied by acute phase response (APR) [1]. This process is manifested by increased concentration of biomarkers—IL-6 and C-reactive protein [2–5], together with activation of the coagulation/fibrinolysis cascade [4, 6, 7], but not blood platelets [8, 9]. Unfortunately, these biomarkers may reflect not only the activity and severity of CU [3, 4], but correlate with a systemic response to infections [10]. In contrast, procalcitonin (PCT) seems to be more specifically associated with the presence of microbial infection, only slightly elevated in rare cases of immune-mediated inflammatory diseases [11–13]. Interestingly, determination CRP and PCT together was superior to CRP alone for diagnosing active or severe inflammatory bowel diseases [14].

So far, there are no available data regarding behaviour of PCT concentration in patients with CU. Characterization of inflammatory response in CU may appear essential for an insight into the pathophysiology of the disease as well as its activity and severity, and diagnosis/management of infections in the course of CU [1]. Therefore, in the present study, serum concentration of PCT has been investigated in patients with CU.

## Method

Twenty-one patients with active CU (median age 36 years) were enrolled in the study.

Urticaria activity score (UAS) according to EAACI/GALEN/EDF guidelines [15] was estimated during 4 days and on the day of blood sampling: (no wheals = 0, 1–10 wheals = 1, 11–50 wheals = 2, >50 wheals = 3) and pruritus intensity (no = 0, mild = 1, moderate = 2, severe = 3). UAS scores: daily (minimum = 0; maximum = 6) and 4 days by adding the daily score values (minimum = 0; maximum = 24). The UAS was graded as follows: mild (0–8), moderate (9–16) and severe (17–24). The study comprised 10 patients with mild and 11 patients with moderate-severe urticaria symptoms as per the 4 days UAS. None of the examined subjects had taken oral corticosteroids within 2 months, or antihistamines within at least 4 days before the study. Routine investigations had been performed to exclude any known causes of the diseases or the concomitant diseases, including dental and laryngological consultations and an autologous serum skin test (ASST).

The control group consisted of 14 sex-, age- and BMI- (<30) matched healthy subjects.

The Ethics Committee of the Medical University of Silesia approved of the study and written, informed consent was obtained from all the subjects participating.

### Blood collection

All blood samples were obtained between 7 and 9 a.m. by antecubital puncture.

### Assay of CRP

Serum CRP concentration was measured by a turbidimetric latex agglutination method (CRP-Latex, BioSystems SA, Barcelona, Spain) with a detection limit of 1.0 mg/l. Elevated serum CRP was defined as higher than 5.0 mg/l.

### Assay of procalcitonin

Serum procalcitonin concentration was measured using an electrochemiluminescence immunoassay (ECLIA) method (Roche Diagnostics GmbH, Mannheim, Germany) with a detection limit of 0.02 ng/mL. Elevated serum PCT concentration was defined as higher than 0.05 ng/ml.

## Statistical analysis

Data were delivered as medians and interquartile ranges. Kruskal–Wallis variance analysis was used for screening differences between the groups. Mann–Whitney *U* test was used to compare data between the patient groups and the healthy controls. Correlation coefficient was obtained by Spearman test. *p* values lower than 0.05 were considered significant.

## Results

### Serum PCT and CRP concentrations

**Fig. 1:**
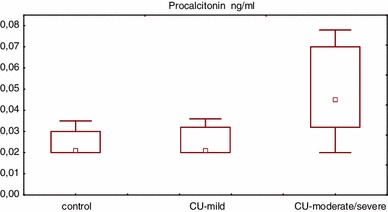
Serum PCT concentration in healthy subjects and chronic urticaria (*CU*) patients with different disease activity. CU-mild vs. control *p* > 0.05, CU-moderate/severe vs. CU-mild vs. control *p* < 0.01

Serum PCT concentration was significantly higher in CU patients as compared with the healthy subjects (median 0.032 vs. 0.021 ng/ml, *p* < 0.05). PCT concentrations were significantly higher in moderate/severe CU patients as compared with mild CU patients and the healthy subjects (median: 0.045 vs. 0.021 vs. 0.022 ng/ml, *p* < 0.01) (Fig 1.). However, only four CU (36 %) patients had PCT slightly elevated above the normal value (defined as higher than 0.05 ng/ml).

Serum CRP concentrations were significantly higher in CU patients as compared with the healthy subjects **(**median and interquartile ranges 9.2 (5.8–14.5) vs. 0.74 (0.3–1.0) mg/l, *p* < 0.0001).

Significant positive correlation was found between serum concentrations of CRP and PCT in CU patients and not in the controls (*r* = 0.69, *p* = 0.008 and *r* = 0.26, *p* = 0.35, respectively).

## Discussion

Our previous data show that circulating levels of CRP and IL-6 were significantly elevated in CU patients and these increases corresponded to the severity and activity of the disease. In addition, a significant association was found between IL-6 and CRP concentrations [3]. In the current study, serum concentrations of PCT and CRP were significantly increased in more severe CU as compared with the healthy subjects and patients with mild disease. However, only a few subjects with severe CU had the values of PCT slightly elevated above the normal laboratory range. In contrast, some values of CRP were highly elevated above the expected range (in some patients higher than 30 mg/l). It seems that significantly increased CRP and PCT concentrations reflect a low-grade non-specific systemic inflammatory response in the course of the disease. Despite extensive investigations we were unable to identify other causes which might be responsible for the increased concentrations.

In healthy subjects, circulating levels of PCT are very low. In viral infections and non-specific inflammatory states, PCT concentration is slightly elevated up to 1.5 ng/ml, but in bacterial systemic infections the level is very high [10, 11, 16].

It is sometimes difficult to distinguish an infection from an exacerbation of the disease in patients with immune-inflammatory disorders associated with elevated serum CRP concentrations [16].

In these situations, PCT offers better specificity than CRP for differentiating between some infections and APR secondary to the diseases activity, regardless of therapy with corticosteroids and immunosuppressive drugs [11, 16]. Taken together, PCT is slightly elevated only in some cases of severe urticaria. Higher PCT level can be observed in local infections in CU patients (own unpublished observations). Therefore, it seems that PCT might reliably differentiate between APR to coincidental infections and secondary to the disease exacerbation/activity.

Of note in our study, a positive correlation was found between levels of CRP and PCT in CU patients and not in the controls. This implies that both PCT and CRP may be regulated by the same mechanisms in CU. It should be indicated that IL-6, a key mediator of acute phase protein synthesis, stimulates not only production of CRP, but also PCT.

Similarly, TNF-alpha and IL-1 beta are able to upregulate PCT synthesis [11, 12, 16, 17]. The site of PCT production in CU is unknown. PCT is produced by different cell types under physiological and pathological conditions, including the neuroendocrine cells, in particular the thyroid C cells, the liver, peripheral blood mononuclear cells, and parenchymal tissues [13, 17, 18].

Unfortunately, we did not study a correlation between IL-6 and PCT in CU patients, which certainly limits our conclusions. In our previous study, a significant association was found between concentrations of CRP and IL-6, and they correlate with the disease activity and severity. PCT behaves in a similar fashion to CRP, thus serum concentration of PCT may reflect, at least in part, the severity/activity of CU.

Currently, it is not clear whether the increased circulating PCT is merely an epiphenomenon or may be related to the pathogenesis of CU. Interestingly, it has been suggested that PCT may enhance inflammatory processes [11]. PCT-like CRP and IL-6 may reflect not only a low-grade systemic inflammatory response in CU, but also may be cooperatively involved in the pathogenesis of the disease.

## Conclusions

Our results show that serum PCT concentrations were increased in more severe CU patients when compared to the healthy controls and mild CU, and that within the CU population there was a significant correlation between levels of PCT and CRP.

Serum PCT concentrations remained within normal ranges in most CU patients and were only slightly elevated in some severe CU cases.

One of the most important aspects of the study is indirect evidence that PCT may have potential utility as a useful marker for the detection of infection in patients with increased CRP resulting from active non-specific urticarial inflammation.
